# Clinical management and prognostic determinants of gallbladder neuroendocrine carcinoma: a single-institutional analysis of 31 cases

**DOI:** 10.3389/fonc.2025.1686515

**Published:** 2025-11-17

**Authors:** Yuting Luo, Yu Zhou, Gang Wang, Saud Ahmad Saad, Dan Jiang, Zhen You

**Affiliations:** 1 Division of Biliary Tract Surgery, Department of General Surgery, West China Hospital, Sichuan University, Chengdu, Sichuan, China; 2 Department of Pathology, West China Hospital, Sichuan University, Chengdu, Sichuan, China; 3 West China School of Medicine, West China Hospital, Sichuan University, Chengdu, Sichuan, China

**Keywords:** gallbladder neuroendocrine carcinoma, platinum-based chemotherapy, survival analysis, prognostic factors, retrospective study

## Abstract

**Background:**

Gallbladder neuroendocrine carcinoma (GB-NEC) is an exceptionally rare and highly aggressive malignancy, accounting for only 0.2% of gastrointestinal neuroendocrine neoplasms and 2.3% of gallbladder cancers. Due to its nonspecific clinical presentation and diagnostic challenges, most patients present with advanced disease at diagnosis, resulting in poor prognosis with median survival typically under 12 months. This study aimed to analyze clinicopathological characteristics and identify independent prognostic factors in GB-NEC patients.

**Methods:**

We conducted a retrospective cohort study of 31 histologically confirmed GB-NEC cases treated at a tertiary referral center between 2015-2024. Comprehensive data including demographic characteristics, tumor markers, pathological features (differentiation, Ki-67 index, invasion patterns), treatment modalities (surgical approach, chemotherapy regimens), and survival outcomes were analyzed. Statistical methods included Kaplan-Meier survival analysis, log-rank tests, and multivariate Cox proportional hazards regression models.

**Results:**

The cohort demonstrated median progression-free survival of 12 months and overall survival of 36 months. Multivariate analysis identified three independent poor prognostic factors: elevated alpha-fetoprotein (AFP) (HR 1.01, p=0.034), mixed neuroendocrine-non-neuroendocrine histology (HR 3.90, p=0.042), and delayed adjuvant chemotherapy (HR 15.62, p=0.006).

**Discussion:**

This study establishes AFP elevation, mixed histology, and delayed chemotherapy as critical determinants of poor prognosis in GB-NEC. Our findings emphasize the importance of early diagnosis, aggressive surgical resection, and timely initiation of platinum-based adjuvant therapy.

## Graphical Abstract

Graphical abstract (Created in https://BioRender.com).

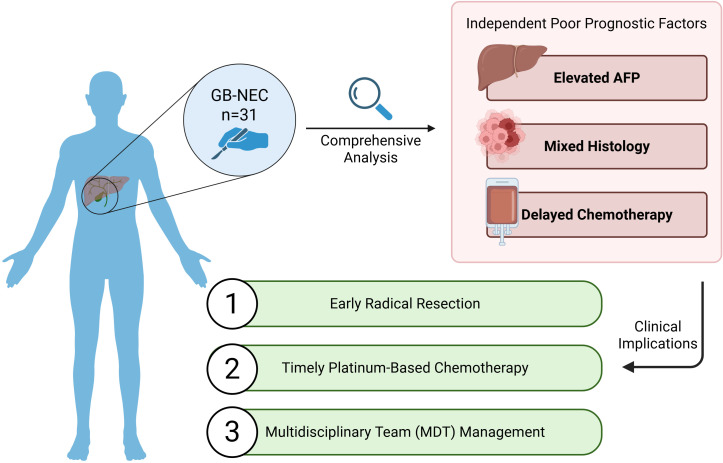

## 1 Introduction

Neuroendocrine carcinoma (NEC) is a poorly differentiated, highly aggressive epithelial tumor with neuroendocrine differentiation features, often diagnosed at advanced stages (1). As a subtype of neuroendocrine neoplasms (NENs), NEC can occur in the digestive system, respiratory system, thyroid, and other organs. Its diagnosis relies on immunohistochemical markers such as chromogranin A (CgA) and synaptophysin (Syn) (2). According to the World Health Organization (WHO) fifth edition classification of digestive system tumors, NENs are further categorized into neuroendocrine tumors (NETs) and NECs, which exhibit distinct molecular characteristics and biological behaviors (3). Although NEC accounts for less than 1% of all malignancies (4), its aggressive nature and poor prognosis have drawn significant attention. Within the gastrointestinal tract, NEC is most commonly found in the rectum, jejunum-ileum, and pancreas (1). Gallbladder NEC (GB-NEC) is particularly rare, representing only 0.2% of gastrointestinal NEC and 2.3% of gallbladder malignancies (5, 6). While adenocarcinoma constitutes over 90% of gallbladder cancers, the clinical characteristics, treatment strategies, and prognostic factors for GB-NEC remain poorly defined (7).

The clinical manifestations of GB-NEC are nonspecific, often including right upper quadrant pain, abdominal distension and jaundice. These symptoms can be mistaken for cholelithiasis, leading to delayed diagnosis (8–10). Current research on GB-NEC is largely limited to case reports, and treatment strategies are typically extrapolated from those for gallbladder adenocarcinoma, lacking specificity. However, a recent large-scale retrospective study of 56 GB-NEC cases—the largest series reported to date in the literature—has provided valuable insights into the pathological characteristics and diagnosis of this disease. While this represents a significant advance, the authors acknowledge that clinical follow-up data remain limited, highlighting a critical area for future investigation (11). Surgery remains the primary treatment modality, with early-stage patients potentially requiring only cholecystectomy, while advanced cases may necessitate extended resection combined with platinum-based adjuvant chemotherapy (12–14). However, since GB-NEC is frequently diagnosed at advanced stages, patient prognosis is extremely poor, with a median survival of merely 8.9 months, significantly lower than that of gallbladder adenocarcinoma (15). Previous studies reported 1-year, 2-year, and 3-year survival rates as low as 20%, 10%, and 0%, respectively (16), with prognostic factors including adjuvant therapy, tumor size and TNM stage (17, 18). Currently, there are no standardized guidelines for the diagnosis and treatment of GB-NEC. To build upon the growing understanding of this disease, particularly the need for comprehensive survival and outcomes analysis, this study retrospectively analyzed clinical data from 31 GB-NEC patients treated at West China Hospital between January 2015 and December 2024, aiming to identify survival-related prognostic factors and provide evidence for clinical decision-making.

## 2 Method

### 2.1 Study cohort

Between January 2015 and December 2024, 1,207 patients underwent surgical treatment for gallbladder cancer at West China Hospital, Sichuan University. Postoperative pathological specimens were examined via histopathological evaluation (hematoxylin and eosin staining, HE) and immunohistochemistry. The antibodies used in this study included Syn, CgA, CD56, cytokeratin 7 (CK7), CK5&6, pan-cytokeratin (pan-CK), p53, retinoblastoma protein (Rb), and Ki-67 (MIB-1). According to the WHO 2022 classification of endocrine and neuroendocrine tumors, gallbladder NECs are poorly differentiated malignancies of biliary epithelial origin with neuroendocrine differentiation features. Histologically, they are classified into small cell type (small cells with hyperchromatic nuclei, scant cytoplasm, arranged in oat cell-like/nested patterns) and large cell type (large cells with prominent nucleoli, abundant cytoplasm, and organoid/trabecular structures), with a Ki-67 proliferation index ≥20%. Immunohistochemical staining confirmed neuroendocrine components in NECs via positive expression of Syn, CgA, or CD56 (at least one marker positive), while epithelial origin was verified through CK7 and pan-CK. Additionally, NECs often exhibit p53 mutations (diffuse strong positivity or null expression on immunohistochemistry) and Rb protein loss (negative Rb expression), both of which aid in NEC diagnosis. Mixed neuroendocrine-non-neuroendocrine tumors (MiNENs) require both neuroendocrine (≥30% of tumor composition) and non-neuroendocrine components (e.g., adenocarcinoma, squamous carcinoma), each meeting their respective diagnostic criteria: neuroendocrine components must fulfill NEC diagnostic requirements, while non-neuroendocrine components (adenocarcinoma or squamous carcinoma) require verification via CK7/pan-CK or CK5&6 positivity, respectively.

**Figure 1:**
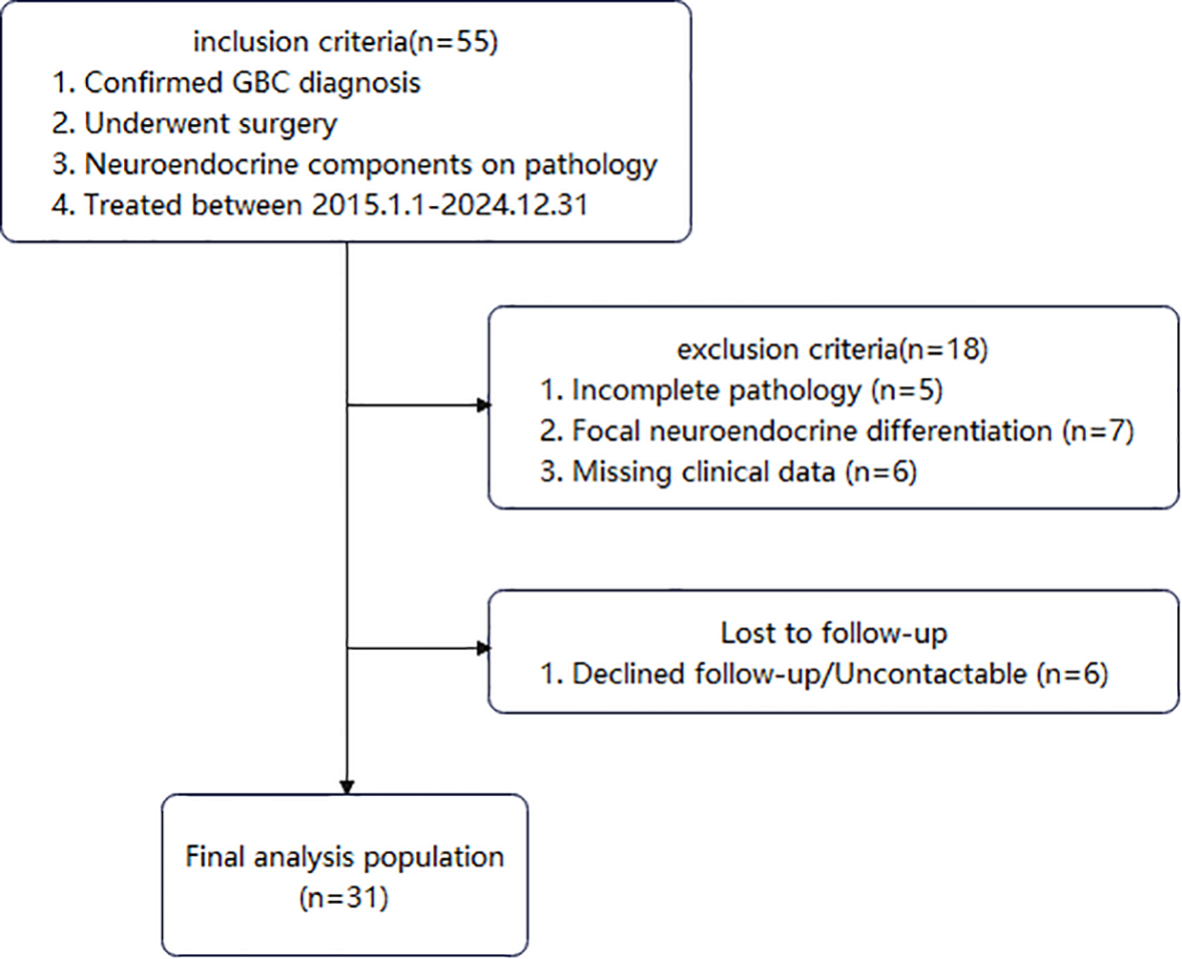
Flowchart of the study.

Among these patients, 55 had pathological findings indicating neuroendocrine components. Patients with incomplete pathology, focal neuroendocrine differentiation, or missing clinical data were excluded. Ultimately, 31 patients diagnosed with GB-NEC were included (**Figure 1**). The study protocol was approved by the hospital’s Institutional Review Board.

### 2.2 Characteristics

Medical records of all 31 GB-NEC patients were retrospectively reviewed, including age, sex, BMI, symptoms, comorbidities, HBsAg status, alkaline phosphatase (ALP), lactate dehydrogenase (LDH), tumor markers (AFP, CEA, CA19-9), and Child-Pugh liver function classification. Pathological features included tumor diameter, liver invasion, liver metastasis, histological type, histological component, lymph node metastasis, lymphovascular invasion (LVI), perineural invasion (PNI), and Ki-67 index. Treatment-related characteristics encompassed surgical approach, neoadjuvant therapy, adjuvant chemotherapy, and radiotherapy. Tumor staging followed the 8th edition American Joint Committee on Cancer (AJCC) staging system.

### 2.3 Follow-up

Postoperatively, patients underwent blood tests and abdominal ultrasound every 3 months in the first year, then every 6 months thereafter. Suspected recurrence or distant metastasis was further evaluated via CT/MRI or PET/CT. Overall survival (OS) was defined as the time from treatment initiation to death, while progression-free survival (PFS) was defined as the time from treatment initiation to first confirmed recurrence. Follow-up ended at patient death or the last follow-up date (April 1, 2025).

### 2.4 Data analysis

Statistical analyses were performed using R software (version 4.2.3). Continuous variables were expressed as mean ± standard deviation (SD) or median (range). Kaplan-Meier curves were generated using GraphPad Prism 9.5 to assess OS and PFS differences in the overall population and subgroups. To ensure sufficient events per variable (EPV) for survival analysis, BMI, N stage, and TNM stage were dichotomized based on clinical relevance and sample distribution. The log-rank test compared survival differences across patient, tumor, and treatment-related characteristics. Univariate and multivariate Cox proportional hazards regression models identified independent risk factors. To avoid information loss in this exploratory study, restricted cubic splines (RCS) were used to test nonlinear relationships, retaining continuous variables. For multivariate analysis, candidate variables were first selected from univariate analyses with P<0.05. Continuous variables were evaluated for multicollinearity using Pearson correlation analysis (with correlation coefficients >0.7 considered clinically significant), while categorical variable associations were examined through χ2 tests. All selected variables subsequently underwent variance inflation factor (VIF) assessment, where VIF values ≥5 indicated problematic collinearity requiring exclusion. This systematic approach ensured the final multivariate model incorporated only statistically independent predictors while maintaining clinical relevance and minimizing overfitting. P<0.05 was considered statistically significant.

## 3 Result

### 3.1 Clinical, pathological characteristics and surgical methods

Complete clinical and pathological data were obtained for all 31 GB-NEC patients, with a median follow-up of 31 months (**Table 1**). The cohort included 21 (67.7%) females and 10 (32.3%) males, aged 37–79 years. The most common symptom was abdominal pain (22, 67.7%), followed by nausea (8, 25.8%) and back pain (7, 22.6%). Five (16.1%) patients were asymptomatic, diagnosed incidentally during routine examinations. Seven (22.6%) had a history of gallstones, and five (16.1%) were HBsAg-positive.

**Table 1:** Clinical and pathological characteristics of 31 patients with GBNEC.

Variables	GBNEC (n=31)
Age, years, median (range)	57 (37–79)
Sex, male/female	10/21
BMI, kg/m², median (range)	23.3% (16.80%-31.22%)
Symptoms	
None symptom, n (%)	5 (16.1%)
Abdominal pain, n (%)	22 (67.7%)
Nausea, n (%)	8 (25.8%)
Back pain, n (%)	7 (22.6%)
Gallstones, n (%)	7 (22.6%)
HbsAg+, n (%)	5 (16.1%)
Serum albumin, g/l, median (range)	42.7 (34.0-49.3)
Total bilirubin, umol/l, median (range)	21.6 (4.7-134.0)
Prothrombin time, s, median (range)	11.3 (9.8-13.3)
Child-Pugh class, A/B	27/4
ALP, U/L, median (range)	173.9 (42.0-977.0)
LDH, IU/L, median (range)	211.9 (141.0-455.0)
AFP, ng/mL, median (range)	11.8 (0.5-268.0)
CEA, ng/mL, median (range)	3.7 (0.4-25.1)
CA19-9, U/mL, median (range)	51.5 (0.6-611.0)
Surgical type, curative/palliative	23/8
primary tumor size (cm), mean ± SD	3.5 ± 2.2
Liver invasion, n (%)	11 (35.5%)
Histological type, LCNEC/SCNEC	23/8
Component, pure/mixed	10/21
PNI, n (%)	21 (67.7%)
LVI, n (%)	24 (77.4%)
KI-67, %, range	50-90
p53, WT/MT	24/7
Rb, WT/MT	25/6
TNM staging	
2A, n (%)	6 (19.4%)
3A, n (%)	4 (12.9%)
3B, n (%)	7 (22.6%)
4B, n (%)	14 (45.2%)

Regarding treatment, 23 (74.2%) patients received radical resection with histologically confirmed R0 margins, while eight (25.8%) received palliative surgery. Among radical resection patients, 11 underwent cholecystectomy with wedge liver resection, and 12 underwent extended resection (at least gallbladder plus liver segments 4b/5). Palliative procedures included cholecystectomy alone (5 patients), cholecystectomy with hepaticojejunostomy (2 patients), and cholecystectomy with liver radiofrequency ablation (1 patient). All patients underwent lymph node dissection for pathological staging.

Pathological and immunohistochemical findings showed a mean tumor diameter of 3.5 cm, with liver invasion in 11 (35.5%), PNI in 21 (67.7%), and LVI in 24 (77.4%) cases. Tumor components were classified as large cell neuroendocrine carcinoma (LCNEC) (23, 74.2%) or small cell neuroendocrine carcinoma (SCNEC, 8, 25.8%). Since MiNEN requires each component to constitute ≥30% of the tumor (1), “atypical mixed NEC” cases in this study were classified based on coexistence with other tumor types: 10 (32.3%) had pure NEC, while 21 (67.7%) had mixed tumors. According to AJCC 8th edition staging, six (19.4%) patients had stage IIA disease, four (12.9%) stage IIIA, seven (22.6%) stage IIIB, and 14 (45.2%) stage IVB. Among 20 patients receiving chemotherapy, 13 received platinum-based regimens.

### 3.2 Postoperative recurrence

**Figure 2:**
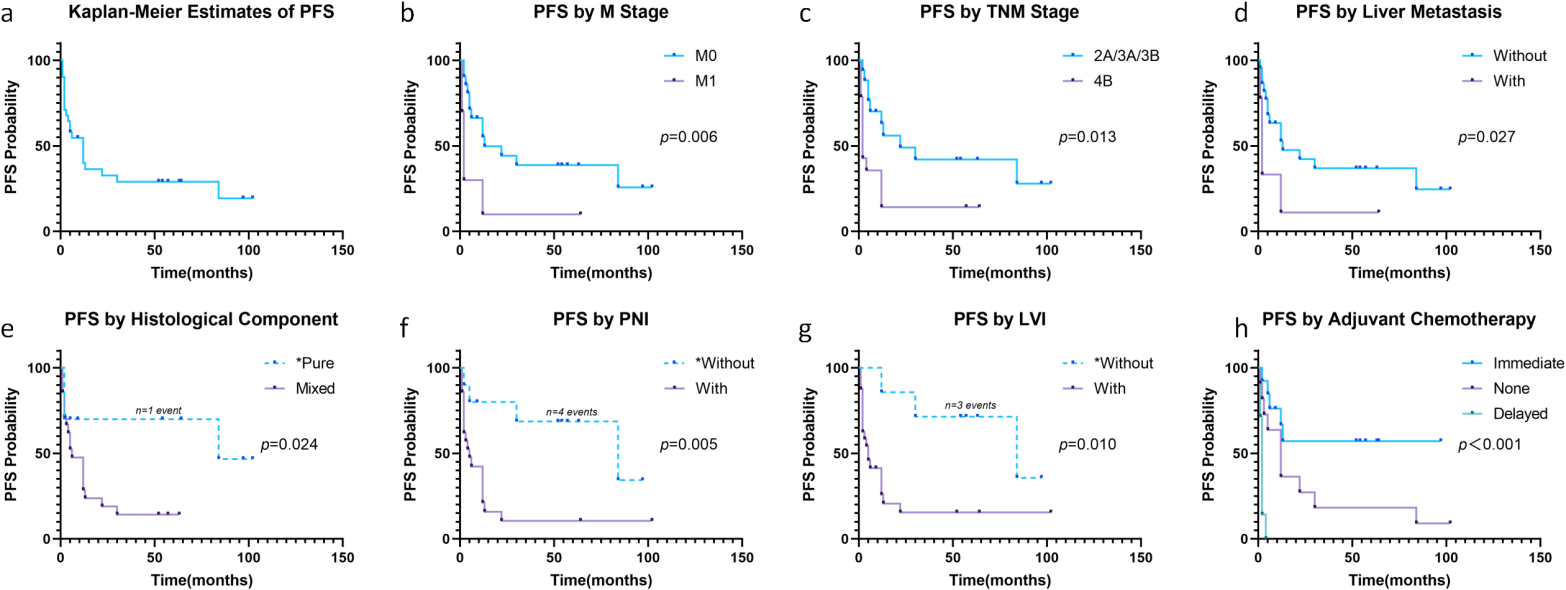
Progression-free survival in all patients **(a)**. Progression-free Survival stratified by M stage **(b)**, TNM stage **(c)**, liver metastasis **(d)**, histological component **(e)**, PNI **(f)**, LVI **(g)** and adjuvant chemotherapy **(h)**. *Caution in interpretation due to <5 events in these subgroups.

During follow-up, tumor recurrence occurred in 22 patients (71.0%; **Figure 2a**). Median PFS was 12 months, with 6-, 12-, and 24-month cumulative recurrence rates of 45.4% (95% CI: 24.6-60.4%), 59.9% (95% CI: 37.6-74.3%), and 67.2% (95% CI: 44.6-80.6%), respectively. Survival analysis revealed significant associations between recurrence and M stage (P = 0.006, **Figure 2b**), TNM stage (P = 0.013, **Figure 2c**), liver metastasis (P = 0.027, **Figure 2d**), histological component (P = 0.024, **Figure 2e**), PNI (P = 0.005, **Figure 2f**), LVI (P = 0.010, **Figure 2g**), and adjuvant chemotherapy (P<0.001, **Figure 2h**). Multivariate Cox regression identified elevated AFP (HR: 1.01, 95% CI: 1.003-1.024, P = 0.013), mixed histology (HR: 3.90, 95% CI: 1.048-14.552, P = 0.042), and delayed chemotherapy (HR: 18.22, 95% CI: 3.561-93.211, P = 0.042) as independent prognostic factors for recurrence (**Table 2**, **Supplementary Table S1**).

**Table 2:** Multivariate Cox regression analysis of progression-free survival and overall survival in all patients.

Variables	PFS			OS		
	Univariate P	Multivariate P	Hazard Ratio	Univariate P	Multivariate P	Hazard Ratio
Total bilirubin						
Per 1 μmol/L increase	0.279			**0.031**	0.062	1.02 (0.999-1.035)
AFP*						
Per 1 ng/mL increase	**0.012**	**0.013**	**1.01 (1.003-1.024)**	**0.017**	**0.034**	**1.01 (1.0001-1.025)**
CEA*						
Per 1 ng/mL increase	**0.007**			0.053		
Surgery type						
Curative	Ref.			Ref.		
Palliative	0.101			**0.004**	0.137	2.57 (0.740-8.932)
Liver metastasis†						
No	Ref.			Ref.		
Yes	**0.021**			0.229		
Component						
Pure	Ref.	Ref.	Ref.	Ref.	Ref.	Ref.
Mixed	**0.041**	**0.042**	**3.90 (1.048-14.552)**	**0.037**	0.100	6.07 (0.710-51.992)
PNI‡						
No	Ref.	Ref.	Ref.	Ref.		
Yes	**0.009**	0.142	2.63 (0.724-9.525)	0.102		
LVI‡						
No	Ref.			Ref.		
Yes	**0.018**			0.082		
M†						
0	Ref.	Ref.	Ref.	Ref.		
1	**0.006**	0.245	1.87 (0.649-5.414)	0.096		
Adjuvant chemotherapy						
Immediate	Ref.	Ref.	Ref.	Ref.	Ref.	Ref.
Delayed	**<0.001**	**<0.001**	**18.22 (3.561-93.211)**	**0.005**	**0.006**	**15.62 (2.239-108.994)**
None	0.070	0.458	1.57 (0.480-5.105)	**0.043**	0.145	3.35 (0.658-17.022)

^*^AFP was selected over CEA for multivariate analysis due to collinearity (Pearson r > 0.7).

^†^M stage was selected over liver metastasis due to collinearity (χ² P < 0.05).

^‡^PNI was selected over LVI due to collinearity (χ² P < 0.05).

Only variables with significant associations in univariate analysis (**Supplementary Table S1**) were included in this multivariate model.

Bold values indicate statistically significant P values and Hazard Ratios (HR).

### 3.3 Survival outcomes

**Figure 3:**
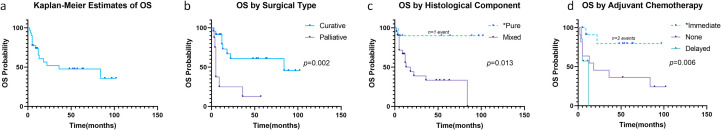
Overall survival in all patients **(a)**. Overall Survival stratified by surgical type **(b)**, histological component **(c)** and adjuvant chemotherapy **(d)**. *Caution in interpretation due to <5 events in these subgroups.

Fifteen (48.4%) patients died during follow-up (**Figure 3a**). Median OS was 36 months, with 6-, 12-, and 24-month cumulative mortality rates of 22.6% (95% CI: 6.4-36.0%), 34.9% (95% CI: 14.4-50.6%), and 48.0% (95% CI: 24.4-64.2%), respectively. Survival analysis showed OS was associated with surgical type (P = 0.002, **Figure 3b**), histological component (P = 0.013, **Figure 3c**), and adjuvant chemotherapy (P = 0.006, **Figure 3d**). Multivariate Cox regression confirmed elevated AFP (HR: 1.01, 95% CI: 1.000-1.028, P = 0.034) and delayed chemotherapy (HR: 15.62, 95% CI: 2.239-108.994, P = 0.006) as independent prognostic factors for OS (**Table 2**, **Supplementary Table S1**).

## 4 Discussion

This retrospective study analyzed 31 GB-NEC patients, revealing a 71.0% recurrence rate (median PFS: 12 months) and 48.4% mortality rate (median OS: 36 months). Univariate analysis identified AFP, M stage, TNM stage, liver metastasis, surgical type, tumor composition, PNI, LVI, and adjuvant chemotherapy as significant prognostic factors. Multivariate analysis further established elevated AFP, mixed histology, and delayed chemotherapy as independent risk factors for recurrence, while AFP and delayed chemotherapy also independently impacted OS.

The 2010 WHO classification of digestive system tumors categorized well-differentiated NETs as G1/G2 and poorly differentiated NETs (G3) as NECs, with mixed adenoneuroendocrine carcinomas (MANECs) representing mixed tumors (19). The 2022 WHO classification distinguished G3 NETs from NECs and introduced mixed neuroendocrine-non-neuroendocrine neoplasms (MiNENs) for tumors containing ≥30% of both components (20). Studies confirm NETs and NECs exhibit distinct survival patterns, necessitating separate consideration (21, 22). Among NECs, colorectal cases show the best survival, while gallbladder/biliary NECs have the worst (23). Preoperative diagnosis of GB-NEC remains challenging, often requiring postoperative pathology (5), and its rarity and poor prognosis complicate management. The management of rare and aggressive neoplasms like GB-NEC is best guided by multidisciplinary teams leveraging established international guidelines. While specific guidelines for GB-NEC are limited, frameworks from the European Neuroendocrine Tumor Society (ENETS) and the National Comprehensive Cancer Network (NCCN) for poorly differentiated neuroendocrine carcinomas of other sites provide valuable direction, emphasizing the critical importance of radical surgery and platinum-based chemotherapy (23, 24). Our findings strongly align with these principles.

Previous studies report varying survival outcomes for GB-NEC: Chen et al. (16) reported a median OS of 3 months, Jiang et al. (17) 16.8 months, and our study 36 months—likely due to higher rates of radical resection and chemotherapy in our cohort. Another study of 34 GB-NEC patients reported 1-, 3-, and 5-year OS rates of 64%, 35%, and 19%, respectively (18), aligning with our findings. While the recent large-scale study of 56 GB-NEC cases has provided valuable pathological insights (11), comprehensive survival data remain limited due to the reported follow-up constraints. High recurrence rates in GB-NEC (6) were also observed.

This study newly associates elevated AFP as a factor associated with poor prognosis in GB-NEC, potentially due to its association with liver metastasis. While most MiNENs share proliferative indices and genomic alterations with NECs/adenocarcinomas (25), our inclusion of all GB-NECs regardless of neuroendocrine component percentage revealed worse recurrence in mixed tumors—a novel finding suggesting increased aggressiveness in mixed cases. Thus, atypical MiNENs with <30% of either component may require refined classification.

Radical resection, particularly R0 resection, is paramount for GB-NEC (26, 27). Although radical surgery prolonged survival in our study (nonsignificant in multivariate analysis), we strongly recommend early radical resection for resectable tumors. The role of adjuvant therapy remains debated: some studies report no survival benefit (26, 28), while others demonstrate significant OS improvement (17, 18, 29). In our cohort, 13/20 chemotherapy patients received platinum-based regimens, with timely chemotherapy significantly improving outcomes versus delayed treatment. Thus, early adjuvant chemotherapy is strongly recommended. This observation is consistent with current clinical understanding of NEC management. For poorly-differentiated neuroendocrine carcinomas like GB-NEC, platinum-based chemotherapy forms the cornerstone of systemic treatment. While no randomized trials have specifically addressed G3 extra-pulmonary NENs, platinum-etoposide combinations remain the regimen of choice based on retrospective evidence, with carboplatin often preferred over cisplatin due to comparable efficacy and better tolerance (30). Beyond first-line treatment, the role of second-line chemotherapy remains limited and requires careful consideration of patient performance status and potential risks versus benefits. Case reports highlight successes with platinum-based regimens (10, 14, 31–36), though some patients still experience rapid progression (37, 38), underscoring the need for multimodal therapy. Multidisciplinary team (MDT) management is essential, particularly for mixed tumors requiring component-specific chemotherapy. Although neoadjuvant chemotherapy (NACT) showed no survival benefit here (limited cases), prior studies suggest tumor downsizing via NACT facilitates resection (39). Biotherapy and adjuvant chemoradiation may also hold promise (40); these modalities should be considered in MDT discussions, warranting further exploration of personalized, multimodal approaches for GB-NEC.

This study has several limitations. Its single-center, retrospective nature and small sample size limit the statistical power for some subgroup analyses. The preoperative diagnosis of GB-NEC was challenging, and insufficient data on biomarkers like neuron-specific enolase (NSE) and CgA precluded their prognostic evaluation. Furthermore, as over half of our patients were still alive at the time of analysis, our current survival data require further maturation. Therefore, we plan to extend the follow-up period for this cohort and continuously enroll newly diagnosed patients in the future. This ongoing effort will be crucial for obtaining more robust survival statistics, increasing the sample size, and ultimately validating our findings with greater accuracy. Moreover, prospective, multi-institutional collaborations will be essential to independently validate our findings, refine risk stratification, and explore the efficacy of novel therapeutic agents for this challenging disease.

## 5 Conclusion

In conclusion, GB-NEC is a highly aggressive malignancy with dismal prognosis. Elevated AFP, mixed histology, and delayed chemotherapy are key prognostic factors for poor outcomes. Preoperative diagnosis remains difficult; thus, heightened clinical awareness, R0 resection combined with early platinum-based chemotherapy, and multimodal therapy are crucial. Multicenter studies incorporating pathological and immunohistochemical profiling are needed to optimize individualized treatment strategies.

## Data Availability

The raw data supporting the conclusions of this article will be made available by the authors, without undue reservation.
