# Supplementary material for: Clinical management and prognostic determinants of gallbladder neuroendocrine carcinoma: a single-institutional analysis of 31 cases
Source: Front Oncol. 2025 Nov 17;15:1686515. doi: 10.3389/fonc.2025.1686515 (PMC12665597; doi:10.3389/fonc.2025.1686515)
Supplement: Supplementary file 1 [file DataSheet1.docx]

*
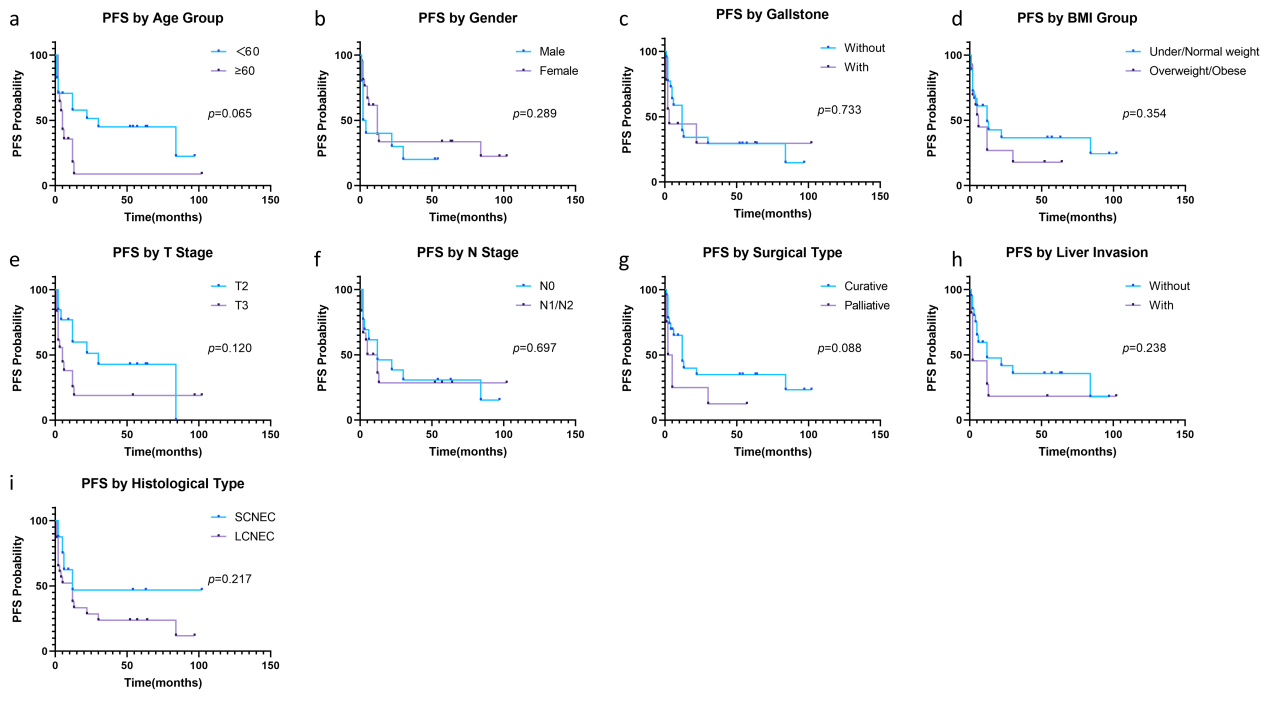
Figure S1: Progression-free Survival* *stratified by age group(a), gender (b), gallstone(c), BMI group (d), T stage(e), N stage (f), surgical type (g), liver invasion(h) and histological type(i).*

*
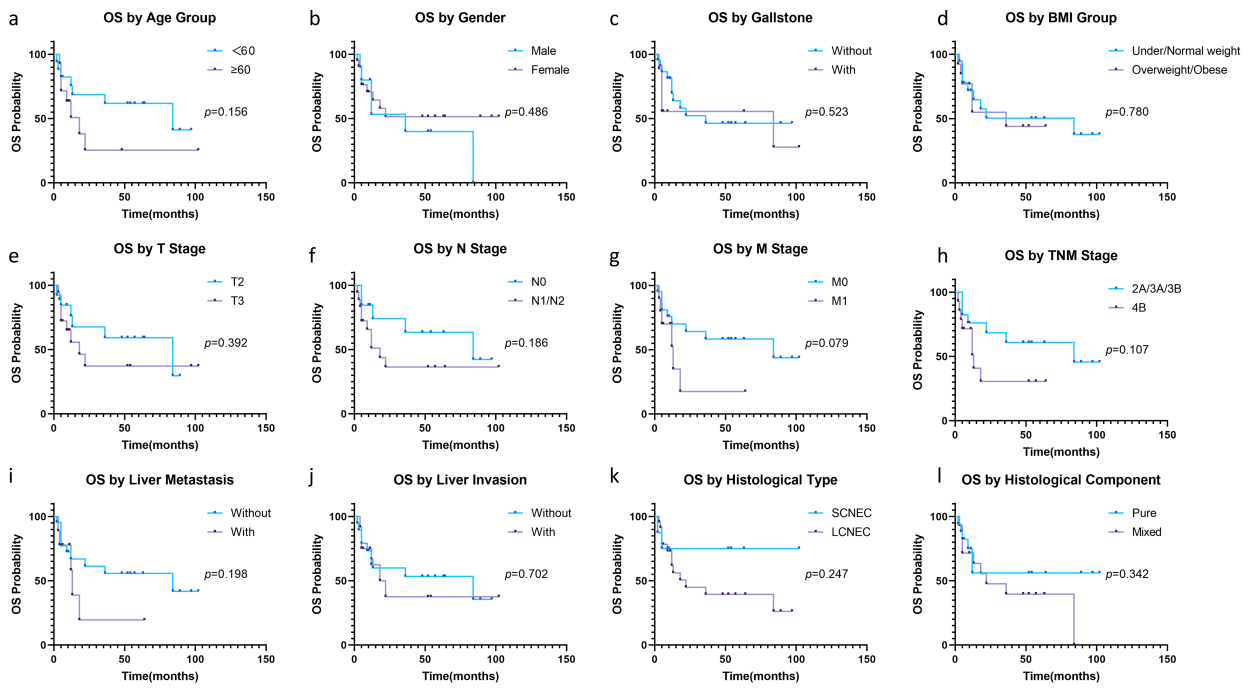
Figure S2: Overall Survival stratified by age group(a), gender (b), gallstone(c), BMI group (d), T stage(e), N stage (f), M stage(g), TNM stage(h), liver metastasis (i), liver invasion(j), histological type(k) and histological component(l).*

Table S1: Multivariate Cox regression analysis of progression-free survival and overall survival in all patients.

| Variables |  | PFS | | |  | OS | | |
| --- | --- | --- | --- | --- | --- | --- | --- | --- |
|  |  | Univariate P | Multivariate P | Hazard Ratio |  | Univariate P | Multivariate P | Hazard Ratio |
| Age |  | 0.071 |  |  |  | 0.547 |  |  |
| Per 1 year increase |  |  |  |  |  |  |  |  |
| Sex |  |  |  |  |  |  |  |  |
| Male |  | Ref. |  |  |  | Ref. |  |  |
| Female |  | 0.313 |  |  |  | 0.488 |  |  |
| BMI |  |  |  |  |  |  |  |  |
| Underweight |  | Ref. |  |  |  | Ref. |  |  |
| Normal weight |  | 0.450 |  |  |  | 0.722 |  |  |
| Overweight |  | 0.366 |  |  |  | 0.646 |  |  |
| Obese |  | 0.192 |  |  |  | 0.809 |  |  |
| Gallstone |  |  |  |  |  |  |  |  |
| No |  | Ref. |  |  |  | Ref. |  |  |
| Yes |  | 0.753 |  |  |  | 0.492 |  |  |
| Serum albumin |  | 0.297 |  |  |  | 0.861 |  |  |
| Per 1 g/L increase |  |  |  |  |  |  |  |  |
| Total bilirubin |  | 0.279 |  |  |  | **0.031** | 0.062 | 1.02(0.999-1.035) |
| Per 1 μmol/L increase |  |  |  |  |  |  |  |  |
| Prothrombin time |  | 0.648 |  |  |  | 0.829 |  |  |
| Per 1 sec increase |  |  |  |  |  |  |  |  |
| Child Pugh |  |  |  |  |  |  |  |  |
| A |  | Ref. |  |  |  | Ref. |  |  |
| B |  | 0.398 |  |  |  | 0.988 |  |  |
| ALP |  | 0.224 |  |  |  | 0.051 |  |  |
| Per 1 U/L increase |  |  |  |  |  |  |  |  |
| LDH |  | 0.594 |  |  |  | 0.211 |  |  |
| Per 1 IU/L increase |  |  |  |  |  |  |  |  |
| AFP* |  | **0.012** | **0.013** | **1.01(1.003-1.024)** |  | **0.017** | **0.034** | **1.01(1.001-1.025)** |
| Per 1 ng/mL increase |  |  |  |  |  |  |  |  |
| CEA* |  | **0.007** |  |  |  | 0.053 |  |  |
| Per 1 ng/mL increase |  |  |  |  |  |  |  |  |
| CA19-9 |  | 0.451 |  |  |  | 0.767 |  |  |
| Per 1 U/mL increase |  |  |  |  |  |  |  |  |
| Surgery type |  |  |  |  |  |  |  |  |
| Curative |  | Ref. |  |  |  | Ref. |  |  |
| Palliative |  | 0.101 |  |  |  | **0.004** | 0.137 | 2.57(0.740-8.932) |
| Primary tumor size |  | 0.129 |  |  |  | 0.669 |  |  |
| Per 1 cm increase |  |  |  |  |  |  |  |  |
| Live invasion |  |  |  |  |  |  |  |  |
| No |  | Ref. |  |  |  | Ref. |  |  |
| Yes |  | 0.207 |  |  |  | 0.964 |  |  |
| Liver metastasis† |  |  |  |  |  |  |  |  |
| No |  | Ref. |  |  |  | Ref. |  |  |
| Yes |  | **0.021** |  |  |  | 0.229 |  |  |
| Histological type |  |  |  |  |  |  |  |  |
| LCNEC |  | Ref. |  |  |  | Ref. |  |  |
| SCNEC |  | 0.234 |  |  |  | 0.267 |  |  |
| Component |  |  |  |  |  |  |  |  |
| Pure |  | Ref. | Ref. | Ref. |  | Ref. | Ref. | Ref. |
| Mixed |  | **0.041** | **0.042** | **18.22(3.561-93.211)** |  | **0.037** | 0.100 | 6.07(0.710-51.992) |
| PNI‡ |  |  |  |  |  |  |  |  |
| No |  | Ref. | Ref. | Ref. |  | Ref. |  |  |
| Yes |  | **0.009** | 0.142 | 2.63(0.724-9.525) |  | 0.102 |  |  |
| LVI‡ |  |  |  |  |  |  |  |  |
| No |  | Ref. |  |  |  | Ref. |  |  |
| Yes |  | **0.018** |  |  |  | 0.082 |  |  |
| Ki 67 |  | 0.733 |  |  |  | 0.733 |  |  |
| Per 10% increase |  |  |  |  |  |  |  |  |
| T |  |  |  |  |  |  |  |  |
| 1 |  | Ref. |  |  |  | Ref. |  |  |
| 2 |  | 0.781 |  |  |  | 0.312 |  |  |
| 3 |  | 0.385 |  |  |  | 0.950 |  |  |
| N |  |  |  |  |  |  |  |  |
| 0 |  | Ref. |  |  |  | Ref. |  |  |
| 1 |  | 0.870 |  |  |  | 0.382 |  |  |
| 2 |  | 0.210 |  |  |  | 0.116 |  |  |
| M† |  |  |  |  |  |  |  |  |
| 0 |  | Ref. | Ref. | Ref. |  | Ref. |  |  |
| 1 |  | **0.006** | 0.245 | 1.87(0.649-5.414) |  | 0.096 |  |  |
| Neoadjuvant chemothrapy |  |  |  |  |  |  |  |  |
| No |  | Ref. |  |  |  | Ref. |  |  |
| Yes |  | 0.19 |  |  |  | 0.892 |  |  |
| Adjuvant chemotherapy |  |  |  |  |  |  |  |  |
| Immediate |  | Ref. | Ref. | Ref. |  | Ref. | Ref. | Ref. |
| Delayed |  | **＜0.001** | **＜0.001** | **18.22(3.561-93.211)** |  | **0.005** | **0.006** | **15.62(2.239-108.994)** |
| None |  | 0.070 | 0.458 | 1.57(0.480-5.105) |  | **0.043** | 0.145 | 3.35(0.658-17.022) |
| Radiotherapy |  |  |  |  |  |  |  |  |
| No |  | Ref. |  |  |  | Ref. |  |  |
| Yes |  | 0.257 |  |  |  | 0.229 |  |  |

* AFP was selected over CEA for multivariate analysis due to collinearity (Pearson r > 0.7).

† M stage was selected over liver metastasis due to collinearity (χ² P < 0.05).

‡ PNI was selected over LVI due to collinearity (χ² P < 0.05).
